# An Explanation User Interface for Artificial Intelligence–Supported Mechanical Ventilation Optimization for Clinicians: User-Centered Design and Formative Usability Study

**DOI:** 10.2196/77481

**Published:** 2026-02-03

**Authors:** Ian-C Jung, Maria Zerlik, Katharina Schuler, Martin Sedlmayr, Brita Sedlmayr

**Affiliations:** 1 Institute for Medical Informatics and Biometry Faculty of Medicine and University Hospital Carl Gustav Carus TUD Dresden University of Technology Dresden Germany

**Keywords:** CDSS, clinical decision support system, XAI, explanation user interface, XUI, usability, formative evaluation, intensive care unit, ICU, explainable artificial intelligence

## Abstract

**Background:**

The integration of artificial intelligence (AI) into clinical decision support systems (CDSSs) for mechanical ventilation in intensive care units (ICUs) holds great potential. However, the lack of transparency and explainability hinders the adoption of opaque AI models in clinical practice. Explanation user interfaces (XUIs), incorporating explainable AI algorithms, are considered a key solution to enhance trust and usability. Despite growing research on explainable AI in health care, little is known about how clinicians perceive and interact with such explanation interfaces in high-stakes environments such as the ICU. Addressing this gap is essential to ensure that AI-supported CDSS are not only accurate but also trusted, interpretable, and seamlessly integrated into clinical workflows.

**Objective:**

This study aimed to evaluate the first iteration of the design and evaluation phase of an XUI for an AI-based CDSS intended to optimize mechanical ventilation in the ICU. Specifically, it explores how different user groups—ICU nurses and physicians—perceive and prioritize explanation concepts, providing the empirical foundation for subsequent refinement iterations.

**Methods:**

A midfidelity prototype was developed using the prototyping software Justinmind, based on existing guidelines, scientific literature, and insights from previous user-centered design (UCD) phases. The design process followed ISO (International Organization for Standardization) 9241-210 principles for UCD and combined qualitative and quantitative feedback to identify usability strengths, design challenges, and role-specific explanation needs. The prototype was evaluated formatively through 2 usability walkthroughs (walkthrough 1: 4 resident physicians and walkthrough 2: 4 ICU nurses), which included guided group discussions and Likert-scale assessments of explanation concepts in terms of understandability, suitability, and visual appeal.

**Results:**

The XUI was structured into 2 levels: a first level displaying high-level explanations (outlier warning and output certainty) alongside the CDSS output, and a second level offering more detailed explanations (available input, feature importance, and rule-based explanation) for users seeking deeper insight. While both user groups appreciated the first level, physicians found the second level of the XUI useful, whereas ICU nurses found it overly detailed. Thus, the structure was able to address the differing needs for explanations. The layered design helped balance transparency and information overload by providing initially concise explanations and more detailed ones on demand. The evaluation further strengthened evidence for role-dependent explanation needs, suggesting that nurses prefer actionable, concise insights, whereas physicians benefit from more granular transparency information.

**Conclusions:**

This study underscores the importance of UCD in designing XUIs for CDSS. It highlights the differing information needs of physicians and ICU nurses, emphasizing the value of involving users early in the development of suitable XUIs. The findings provide practical guidance for designing layered, role-sensitive explanation interfaces in critical care and form the basis for future iterative evaluations and experimental studies assessing their impact on decision-making and clinician trust.

## Introduction

### Background

Artificial intelligence (AI) is expected to benefit the medical domain with improvements in patient care and reduced workload for medical personnel. AI models are increasingly proposed as a knowledge or inference base of clinical decision support systems (CDSSs) to achieve this. These AI-based CDSS are considered well-suited for use in the intensive care unit (ICU) due to the amount of available documented data in the ICU, but the transfer of these systems into clinical practice is still lacking [1]. AI models for which it is impossible to directly infer why they generate an output due to their complexity are called black-box models. The black-box nature of these models poses a barrier to adoption in the medical domain, as it makes it difficult for health care providers to understand, trust, or accept these models [2-4]. According to the EU (European Union) AI Act, AI-based CDSS are classified as high-risk AI systems [5]. For high-risk AI systems, the act requires the possibility of human oversight and the provision of appropriate interfaces for this supervision (EU AI Act, Chapter III, Article 13 and 14) [6]. Approaches from the field of explainable AI (XAI) are a solution to these barriers [7-11]. XAI provides additional information to the output of an AI model to make model reasoning more transparent through explanation user interfaces (XUIs). In the medical domain, XAI has been shown to influence users’ trust [12,13], the perceived usefulness [14] or value [13] of a system, the medical professionals’ confidence in decisions [14], and decision-making [15]. XAI research focuses on creating, optimizing, and evaluating XAI algorithms. Explanations are often designed to meet the needs of (X)AI developers while neglecting the needs of clinical end users [16]. Recently, calls for a user-centered design (UCD) of XUIs have been raised in the medical informatics domain [16-20]*.*

The objective of this paper was to conduct the initial design and evaluation phases of the UCD process for an XUI of an AI-based CDSS intended for use in the ICU. As a use case, we selected the provision of recommendations for ventilation parameter settings in patients receiving mechanical ventilation in the ICU. Patients may undergo mechanical ventilation using ventilators—machines that assist breathing by delivering air into the lungs when they cannot breathe adequately. Another form of life support is extracorporeal membrane oxygenation, which bypasses the lungs—and sometimes the heart—by circulating the patient’s blood through an external machine that oxygenates the blood and extracts carbon dioxide from it. To enable the continuous optimization of a patient’s ventilation, a CDSS must be able to recommend a range of parameters. In mechanical ventilation, these include, for example, the ventilator mode, which defines the overall strategy and level of mechanical support; tidal volume, which specifies the amount of air delivered per breath; and respiratory rate, which determines the number of breaths per minute.

The development of such AI-based CDSS is being carried out within the IntelliLung project, a European Union–funded initiative aimed at improving ventilatory care and reducing the workload of clinical staff [21]. The CDSS will generate recommendations for both mechanical ventilation and extracorporeal membrane oxygenation settings. The research presented in this paper used the IntelliLung CDSS as a real-world use case to anchor the design in clinical practice. However, the design is independent of any project-specific AI algorithm decisions and instead focuses conceptually on XUI design for such a CDSS. As Mohseni et al  [22] proposed, this type of research can be conducted before selecting and developing the AI algorithms and corresponding XAI methods.

### State of the Art

Although much research on XAI focuses on the health care domain [23], to the authors’ knowledge, no paper explicitly addresses the UCD of XUIs for AI-based CDSS aimed at optimizing continuous mechanical ventilation in the ICU. Several papers report on the use of AI algorithms and XAI methods from a technical perspective, for example, to predict the ventilation duration for patients with acute respiratory distress syndrome [24], to predict the disease progression of patients with acute respiratory distress syndrome [25], to predict the ventilator support for patients with COVID-19 [26], and to generate ventilator setting recommendations [27]. Hughes et al [28] report on the context and work process analysis for CDSS supporting the weaning process in the ICU to inform the overall CDSS design for weaning support, but do not focus on optimizing continuous ventilation or designing XUIs for the CDSS.

A limited number of papers address the UCD of XUIs in the ICU [14,29-31]. A preprint by Clark et al [29], published after this study concluded, investigates user requirements for using XAI in CDSS, without focusing on a specific ICU use case. Nagendran et al [30] demonstrated the feasibility of eye-tracking analysis to assess physicians’ interactions with an XUI for an AI-based CDSS, although not focused on ventilation, in a simulated ICU setting. Barda et al [31] designed and evaluated an XUI for an AI-based CDSS that predicts mortality risk in a pediatric ICU. The acceptance of an XUI for a sepsis treatment CDSS by ICU clinicians was investigated by Sivaraman et al [14]. Outside the ICU setting, Schoonderwoerd et al [32] investigated pediatricians’ perspectives on different explanation concepts for a diagnostic CDSS.

### Previous Research

The UCD process was conducted in accordance with DIN (Deutsches Institut für Normung) EN (Europäische Norm) ISO (International Organization for Standardization) 9241-210 [33] to design the XUI for an AI-based CDSS that recommends ventilator settings. This paper focuses exclusively on the first iteration of the design and evaluation phases of the UCD process. These phases rely on the results of the context analysis and the specification of user requirements. The results of the first 2 phases of the UCD process are summarized to provide the necessary context.

During the context analysis, the intended use characteristics of the CDSS were defined as (1) a stand-alone CDSS, which should not replace the ventilation monitor, and (2) a CDSS that would be displayed on a stationary tablet next to the ventilation monitor of an individual patient. The identified main user groups of this CDSS were resident physicians and ICU nurses with the authority to change the patient’s ventilator settings. An initial investigation into user requirements revealed conflicting opinions among future users about the importance of explanations [34]. These results led to a follow-up investigation into users’ preferences for explanation, to identify which explanations to include in a first design iteration. Seventeen explanation concepts, ranging from less complex ones such as available input information to more sophisticated ones such as counterfactual explanations, were presented to the users as low-fidelity mockups in a questionnaire. This step was performed before this study to attain a manageable number of explanation concepts for the first design iteration and a formative evaluation. The selection of explanation concepts for this first iteration of the XUI was based solely on user preferences, rather than on commonly used explanation concepts. Users selected the explanation concepts, output certainty, and outlier warning to be displayed alongside the CDSS recommendations. The output certainty concept informs users about the CDSS’s confidence level in its recommendation. The outlier warning alerts users if one or more input parameter values are highly unusual. Three additional explanation concepts were selected but were not designated to appear directly next to the CDSS output: available input, feature importance, and rule-based explanation. The available input explanation concept informs the user which parameter values were available when generating the recommendation. The feature importance explanation concept shows users how each parameter influenced the recommendation. The rule-based explanation concept presents simplified rules in text or decision tree format, approximating how the recommendation was calculated. These findings served as the foundation for the XUI design phase.

### Study Scope and Research Question

#### Overview

The underlying research aim of this paper is threefold to (1) design midfidelity mockups of the XUI, focusing on its overall structure and different design variants for the explanation concepts, (2) give users an impression of the XUI without fully designing each explanation concept in detail, and (3) gather early formative feedback from future users to support iterative refinement of the XUI in subsequent design phases. Therefore, this study does not aim to deliver a finalized XUI design, nor does it seek to conduct a comprehensive summative evaluation of the XUI, as this will be the focus of future iterations.

The following research questions, aligned with the design and evaluation phases, were developed to guide the investigation.

#### Design Phase

The design phase focused on generating design options for the XUI, with the following research question:

- RQ1: How should the XUI of an AI-based CDSS for mechanical ventilation optimization in the ICU be designed?

#### Evaluation Phase

The evaluation phase focused on collecting early user feedback from clinicians, with the following research questions:

- RQ2: What are clinicians’ perceptions of the proposed XUI of the AI-based CDSS for mechanical ventilation optimization?
- RQ3: What XUI design improvements should be addressed in subsequent design iterations?

## Methods

**Figure 1:**
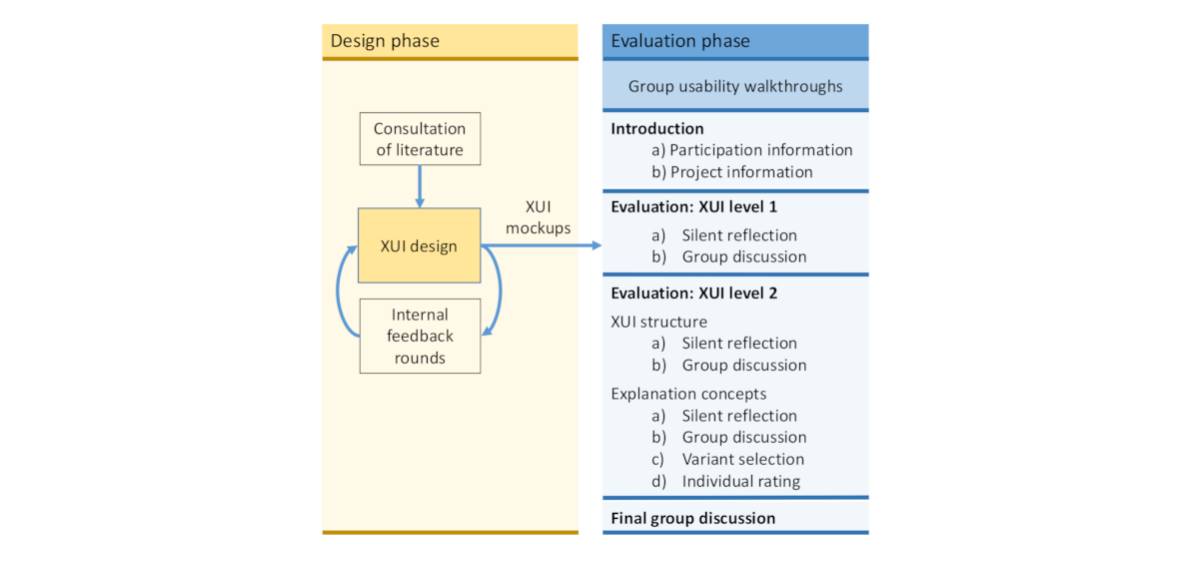
Overview of the methods used during the first iteration of the design and evaluation phases of the explanation user interface (XUI).

Figure 1 provides an overview of the methods used in each XUI development phase.

### XUI Design Phase

The design phase began with the development of a foundational knowledge base for the XUI. Relevant literature, such as norms and design guidelines, was reviewed. For this purpose, the ISO 9241 standard series on human-system interaction and human-centered design was reviewed. This foundation was expanded through a scoping review and the development of design recommendations for the user-centered XUI design for CDSS [35]. Searches for research papers related to the topic of XUIs for AI-based CDSS for the ventilation of patients in the ICU found no relevant results before the design of the XUI.

Midfidelity XUI mockups were developed using the Justinmind [36] prototyping software, based on the knowledge foundation. The design of the mockups was intended to direct users’ attention to elements relevant to RQ2 and RQ3 and to prevent users from focusing on aesthetic aspects such as the color of the XUI. Therefore, mockups were designed with the following characteristics: (1) realistic variable names and content to support user familiarity; (2) realistic-looking data, for which synthetic data points were generated for each displayed input variable with a large language model (Claude Sonnet 3.5 [37]); (3) minimal use of color, used selectively to highlight system functionality; and (4) simple interactions (eg, navigation between interface levels).

A main screen for the CDSS was designed to present the XUI within its intended system context and to help users envision the future context of use for the XUI. The basic structure of the XUI and the design variants for the explanation concepts were developed based on the list of selected explanation concepts and their intended level within the XUI, which had been defined during the requirements specification phase of the UCD process. The mockups were designed iteratively: the first author created initial drafts, and members of the author team with usability expertise provided verbal feedback for improvements during multiple group meetings.

The design of the individual explanation concepts was oriented toward typical design elements used for these explanation concepts in the XAI literature. This decision was made to provide the users with a realistic representation of common explanation concepts and to keep the focus on the goal of this first evaluation iteration. We opted not to apply a more elaborate design strategy to the explanation concepts in this iteration; this will be addressed in later iterations, which typically focus on aesthetics and detailed design.

### XUI Evaluation Phase

In the evaluation phase, formative feedback was gathered from ICU physicians and nurses, focusing on the XUI’s structure and explanation concepts. A formative evaluation was chosen to collect insights for improving the CDSS and understanding users’ initial impressions of the CDSS. Given resource constraints—such as limited availability of clinical experts and their high workload—a pragmatic approach was taken. This evaluation is reported according to the STARE-HI (Statement on the Reporting of Evaluation studies in Health Informatics) guidelines to ensure consistency and standardization [38]). For the STARE-HI reporting checklist, refer to Multimedia Appendix 1 and [39].

### Study Design

As a formative evaluation, 2 group usability walkthroughs were conducted—1 for each main user group (physicians and ICU nurses). A semistructured approach was followed, guided by a predefined protocol. Each walkthrough included (1) a presentation of the XUI, (2) individual reflection time, (3) guided group discussions, and (4) a quantitative assessment (QA) of the explanation concepts.

The walkthrough groups were separated by user group. This separation accounted for differing levels of project involvement and aimed to minimize hierarchical bias, particularly the risk of physicians influencing ICU nurses during discussions. The walkthroughs were moderated by a researcher with usability expertise, who introduced the mockup, guided the discussion using predefined questions focused on information retrieval, overall interaction, and the structure of the XUI, and asked follow-up questions where necessary. A second researcher took notes to ensure thorough documentation.

### Sample Size and Recruiting Process

The target was to recruit 4 to 5 participants per user group, as small sample sizes are considered acceptable for formative evaluations in medical informatics [40], and sample size requirements can be adjusted based on system-specific characteristics [41].

Participants were recruited using convenience sampling. For the physician group, invitation emails were sent to 5 physicians in the same research group, which was involved in the IntelliLung project. Four physicians accepted the invitation. To increase the chances of successful recruitment of ICU nurses, 1 of the cooperating physicians identified an interested contact person among the nursing staff in 1 of the ICUs at the same hospital. The contact person informally inquired about his or her peers’ interest in participating in the study. After the nurses were recruited, the contact person shared the contact details with the researchers. To be eligible to participate in the study, all participants were required to have at least 3 months of ventilation experience (demonstrated through working experience in the ICU or research activity in the field of mechanical ventilation). No compensation was paid for participation in the group usability walkthrough.

### Walkthrough Process

The details of the physician walkthrough are described in this section. Any predefined process deviations for the nurse walkthrough are reported in the section “Deviations for the Nurse Group Usability Walkthrough.”

At the start, participants received in-depth information about their rights and the purpose of the usability walkthrough. Afterward, the moderator presented the CDSS use case and the main screen of the CDSS. The XUI mockup evaluation then began. The 2 XUI levels were evaluated sequentially, reflecting their hierarchical structure.

The evaluation began with the main CDSS screen, which included XUI level 1, displayed on a monitor. The moderator provided a verbal introduction. Participants were then given time to observe and reflect on XUI level 1 silently. The reflection was followed by a group discussion guided by the question: “Is the presentation of outlier warning and system certainty on the main screen sufficient?”

The XUI level 2, a subordinate screen providing additional explanation concepts, was evaluated in 2 steps. In the first step, the participants assessed the overall structure of the level. The XUI level 2 was shown, and the participants silently analyzed it. Afterward, the group briefly discussed and then collectively answered the following questions: “In which part of the interface do you find explanations for the recommendation?” and “How do you switch between the explanations?”

In a second step, the individual explanation concepts in XUI level 2 were assessed using the following steps:

- The initial version of the explanation concept was shown, and the participants silently observed and analyzed each explanation concept.
- The group discussed the explanation concept and then provided a collective answer to the questions (Table 1). Whether the group answered the questions correctly was documented.
- Alternative design variants of each explanation concept were presented. The group discussed these variants and selected their preferred variant.
- Each participant then individually assessed the selected version on Likert scales regarding the following 3 dimensions (ie, QA-1, QA-2, and QA-3) with the following anchors:
- QA-1 (ease of understandability): “I find this explanation concept easy to understand.”
- QA-2 (suitability): “I find this explanation concept suitable for everyday clinical work.”
- QA-3 (appealingness): “I find this explanation concept appealingly visualized.”
- Strongly Disagree, Disagree, Neutral, Agree, and Strongly Agree
- Additional options: no answer and free-text comments

**Table 1:** List of questions and items used to evaluate each explanation concept.

Explanation concept	Group discussion questions
Available input (AI)	AI-1: What information do you get from this view? AI-2: In which sections were not all values available when the recommendation was generated? AI-3: How would you get more detailed information on input values? AI- 4: How should the sections be sorted in the “available input” area? Version A (static order): (1) in the presented order and (2) in a different order. Version B (dynamic order): areas with missing values at the top? If Version A was selected, would you prefer (1) or (2)?
Feature importance (FI)	FI-1: What information do you get from this view? FI-2: Which parameter had an importance of 80% for generating the recommendation? FI-3: Where would you have to click to display the importance of more parameters?
Rule-based explanation (R)	R-1: What information do you get from this view?

The complete version of the instrument is provided in Multimedia Appendix 2.

The group usability walkthrough concluded with a general discussion focusing on the following guiding questions: “Should there be more or fewer explanation concepts presented?” and “Are there any missing explanation concepts?” (The physicians had access to previous presentations of other explanation concepts before the group usability walkthrough).

### Deviations for the Nurse Group Usability Walkthrough

To accommodate the ICU nurses’ limited previous exposure to the project, the walkthrough session was adapted to include a more comprehensive introduction to CDSS and AI concepts. Insights from the physician group informed minor refinements to the mockups. Elements that had proven consistently irrelevant or confusing across previous sessions were excluded to maintain focus, while still allowing nurses to contribute unique, user-specific feedback.

Furthermore, in the final group discussion, the nurses were verbally introduced to the possibility of additional explanation concepts (since they had not previously been exposed to other types, such as counterfactual explanations). They were also asked whether the information presented in level 1 of the XUI was sufficient.

### Analysis Approach

#### Qualitative Analysis of Group Discussions

After each group usability walkthrough, the first author combined the notes from both researchers. The second researcher reviewed the merged version, and any disagreements were discussed and resolved jointly. The first author then categorized the notes by XUI level and discussion questions and organized them by content. No predefined coding scheme was used; categories emerged inductively based on the content of the group discussions. Summarizing bullet points were translated into English, as both group usability walkthroughs had been conducted in German, the participants’ native language. The second researcher reviewed the final summary. No verbatim quotes were recorded during documentation of the group usability walkthroughs.

#### Quantitative Data Analysis

The QAs (QA-1, QA-2, and QA-3) of each explanation concept were analyzed purely descriptively due to the limited number of responses per user group. For each explanation concept and user group combination of the QAs (QA-1, QA-2, and QA-3), the number of responses, the number of “No Answer,” the median, and the IQR are reported. In addition, all responses for the items are presented visually as horizontally oriented stacked bar plots.

### Ethical Considerations

This study was conducted in accordance with ethical research principles. It involved low-risk, formative group usability walkthroughs as a qualitative survey or interview method in which clinician participants were shown interface mockups and asked structured questions about usability, understandability, and suitability. No medical interventions were performed, no patient data were collected, and the study did not constitute epidemiological research. In line with the institutional guidance of the Ethics Committee of TU Dresden for such non-interventional survey or interview studies, no IRB or REB approval was sought [42]. All procedures were carried out in accordance with the principles of the Declaration of Helsinki. Informed consent was obtained from all participants after they received detailed information about the study purpose, the walkthrough procedure, data handling, and their rights. Participation was voluntary, and participants could withdraw at any time without consequences. Participants were not compensated for their participation. Privacy and confidentiality were safeguarded by collecting and storing data in pseudonymized form; only non-sensitive, profession-related participant information was collected, no identifying information is reported, and access to raw data was limited to the research team.

## Results

### XUI Design Phase: Structure of the XUI

**Figure 2:**
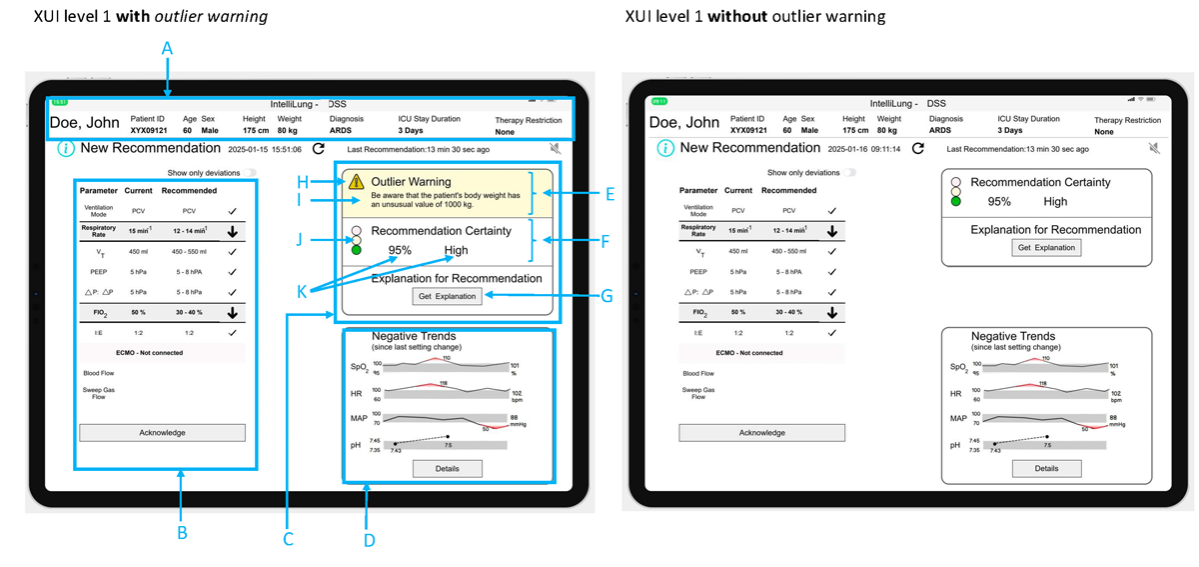
Main screen of the clinical decision support system (CDSS), showing level 1 of the explanation user interface (XUI) in the top-right section. Two versions are shown: the left screen includes the outlier warning, and the right screen does not.

The design phase produced a 2-level XUI structure (XUI level 1 and XUI level 2). XUI level 1 is positioned on the main screen of the CDSS, adjacent to the system’s recommendations. Figure 2 shows the mockup for the CDSS main screen, which contains a header with basic patient information (A in Figure 2), the recommendation (B in Figure 2), XUI level 1 (C in Figure 2), and a trend view of important parameters (D in Figure 2). XUI level 1 includes the explanation concept outlier warning (E in Figure 2), consisting of an icon with a header (H in Figure 2) and an information text (I in Figure 2); the explanation concept certainty information (F in Figure 2), for which the certainty level is presented as a traffic light (J in Figure 2), a number, and a category label (K in Figure 2); and a button (G in Figure 2) that links to XUI level 2.

**Figure 3:**
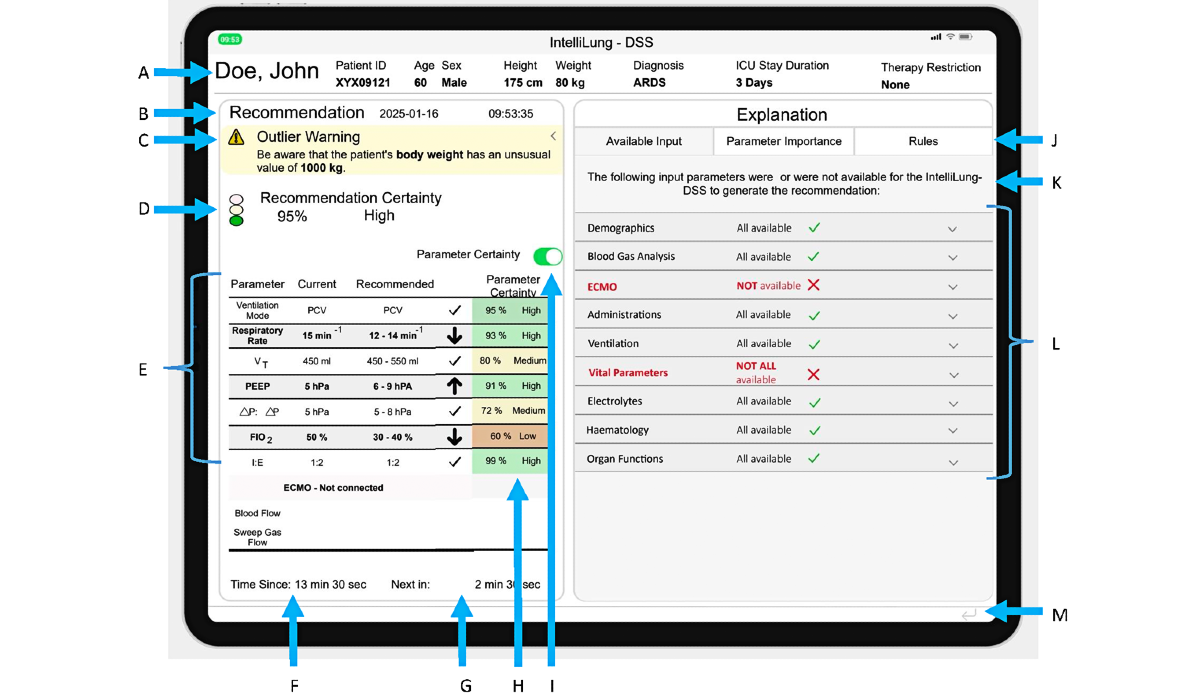
Level 2 of the explanation user interface (XUI) with a description of its content. A: header with basic patient information; B: identification information for the recommendation; C and D: repetition of explainable artificial intelligence (XAI) concepts from the first level; E: repetition of the clinical decision support system (CDSS) recommendation for ventilator setting; F and G: information on when the last recommendation was generated and when the subsequent recommendation will be generated; H: certainty information for each recommended ventilator setting; I: toggle button to show or hide H; J: section for the presentation of explanation concepts, with tabs for selecting specific explanation concepts; K: short sentence introducing the explanation concept; L: space for the specific explanation concepts; M: link to the first level of the XUI. ARDS: acute respiratory distress syndrome; DSS: decision support system; ECMO: extracorporeal membrane oxygenation; FIO2: fraction of inspired oxygen; ICU: intensive care unit; PCV: pressure-controlled ventilation; PEEP: positive end-expiratory pressure; VT: tidal volume; ΔP: driving pressure.

Figure 3 shows XUI level 2, which includes a header with basic patient information, the CDSS recommendation, a repetition of the explanation concepts from level 1, and an expanded panel on the right side for displaying explanation concepts in more detail, one explanation concept at a time. The following explanation concepts were used in level 2: available input, feature importance, and rule-based explanation. The users can switch between explanation concepts in the expanded section using tabs.

### Design of Explanation Concepts

#### Outlier Warning

In XUI level 1, the outlier warning explanation is positioned at the top (E in Figure 2). If an outlier is detected in the input variables, a yellow warning triangle containing an exclamation mark is presented next to the heading “outlier warning.” The heading is followed by text naming the parameter and value that triggered the outlier warning. When an outlier is detected, the background of the outlier warning section is highlighted in yellow. Two options were created for the case that the outlier warning was not triggered: one that hides the outlier warning section entirely, and another that displays the message “No Outlier Warning.” The outlier detection logic was intentionally left undefined at this stage, as the study focused on evaluating conceptual explanation designs within the XUI. The specific detection mechanism will be developed and validated in subsequent iterations.

#### Output Certainty

Below the outlier warning section, the XUI presents the output certainty (F in Figure 2). On the left side of the output certainty section, a traffic light icon indicates the system’s certainty level. This is followed by a percentage value and a categorical label representing the system’s certainty.

#### Available Input

**Figure 4:**
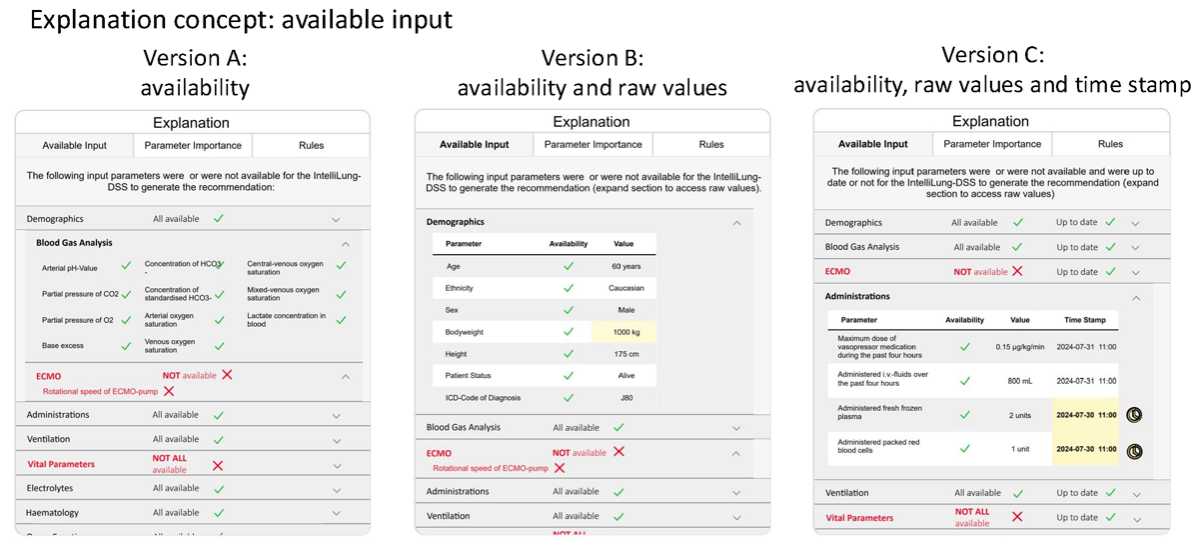
Three versions of the available input explanation concept. Version A: availability information organized in categories. Version B: availability information extended with the values of the parameters. Version C: availability information and parameter values extended with a time stamp for parameter value collection. CO2: carbon dioxide; DSS: decision support system; ECMO: extracorporeal membrane oxygenation; HCO3: bicarbonate; ICD: International Classification of Diseases; O2: oxygen; pH: potential of hydrogen.

Figure 4 provides 3 design versions of the available input explanation concept. The versions vary in how much detail they present to the user.

The input parameters are organized into categories in Version A (Figure 4). Each category is displayed as an expandable section with a labeled heading. Within each category, parameter names are displayed in a matrix format, each accompanied by an icon indicating availability (a green checkmark for available parameters; a red cross for unavailable ones). Unavailable parameters were also highlighted in red text. If any parameters in a category (eg, “Vital Parameters”) were missing, the category heading appeared in bold red text, and a red cross icon with explanatory text was added to the top of the category section.

Version B (Figure 4) extends Version A by including the values of all available parameters. Each category is presented as a table with 3 columns: parameter name, availability status, and parameter value. Cells containing values that triggered an outlier warning are highlighted in yellow.

Version C (Figure 4) further builds on Version B. Each table is expanded with a fourth column displaying the time stamp of when the parameter value was recorded. If the recorded time stamp falls outside a predefined range, the cell is highlighted in yellow, and a yellow clock icon appears next to the row to indicate outdated data.

#### Feature Importance

**Figure 5:**
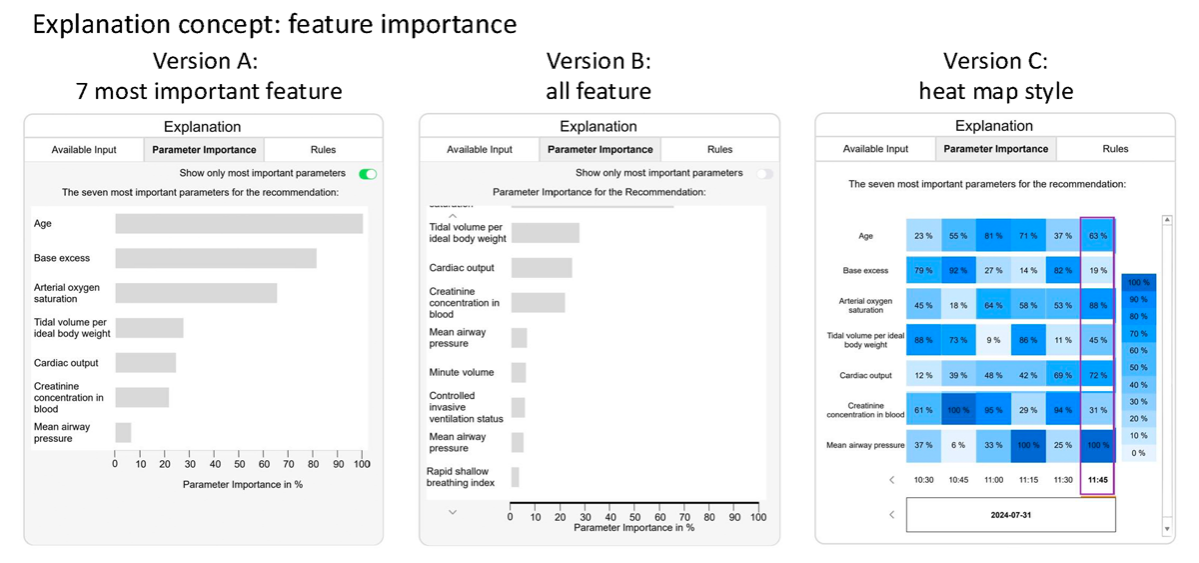
Three versions of the “feature importance” explanation concept. Version A: vertical bar plot of the 7 most important features. Version B: vertical bar plot showing the feature importance of all features. Version C: feature importance displayed as a heat map–style history in table format.

Figure 5 provides 3 versions of the feature importance explanation concept. In the mockup, the explanation concept is labeled as “Parameter Importance” to avoid the necessity of pre-existing knowledge of the term feature in the context of AI for clinicians. This change in the name did not influence the information presented in the explanation concept. The 3 versions differ in the level of detail they provide.

Version A (Figure 5) displays the top 7 features in a vertical bar plot, ranked by importance.

Version B (Figure 5) uses the same bar plot format but includes all available features. The user can scroll through the bar plot vertically and toggle between Versions A and B.

Version C (Figure 5) provides the 7 most important features of the current recommendation in a heat map history, which is designed as a table. The rightmost column contains the feature importance values for the current recommendation. The cell background color corresponds to the value of the feature importance presented in the legend. The preceding columns represent the feature importance for previous recommendations. Clicking on a column updates the recommendation displayed in the left-side panel of the XUI (B in Figure 3).

#### Rule-Based Explanation

**Figure 6:**
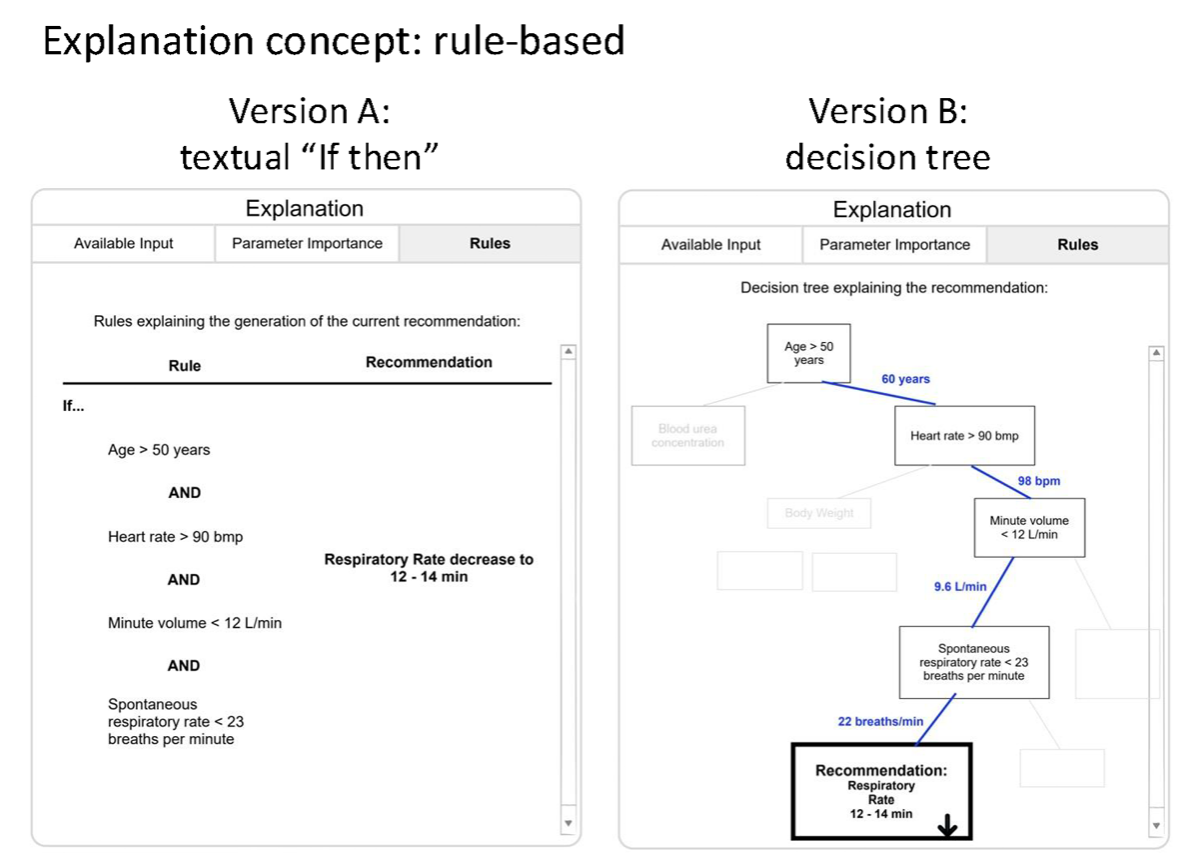
Different versions of the rule-based explanation concept. Version A: textual representation as an “if-then” phrase. Version B: graphical representation as a decision tree.

Two versions of the rule-based explanation concept were designed (Figure 6).

Version A (Figure 6) provides the explanation concept as an “if-then” rule in text format. Version B (Figure 6) uses a visual decision tree to convey the same logic. The decision tree highlights the path to the recommendation with blue lines.

### XUI Evaluation Phase

#### Participants Description

The group usability walkthroughs were conducted with 8 health care professionals from a German university hospital, including 4 ICU physicians (4 male) and 4 ICU nurses (3 male and 1 female). All physicians participated in the preceding UCD process phases of the IntelliLung CDSS interface. In contrast, the recruited ICU nurses had no previous exposure to the IntelliLung CDSS. The recruited physicians were all anesthesiology residents with clinical and research experience in mechanical ventilation. The ICU nurses had at least 9 years of experience working in the ICU. All nurses answered a question describing their experience selecting ventilator settings for patients as “a lot” and a question regarding their experience with AI-based CDSS as “non“ (n=1) and “little“ (n=3) on 4-point scales with the following answer options: “non,” “little,” “moderate,” “a lot.” The physicians were not formally asked about their AI knowledge, but some degree of knowledge can be assumed due to their participation as clinical experts in the development of the IntelliLung AI model.

#### Evaluation: XUI Level 1

Both user groups appreciated the content in XUI level 1 and favored a concise presentation format. However, the groups differed in their preferences regarding how the information should be presented. Table 2 provides a detailed summary of findings.

**Table 2:** Insights explanation user interface (XUI^a^) level 1 evaluation.

XUI part	Physician walkthrough	Nurse walkthrough
Overall assessment (level 1 XUI)	Preferred a simple presentation with minimal content.	Found the information on level 1 important.
Outlier warning	Terminology improvement: suggested renaming the concept to “Input Warning” to avoid confusion with clinical outliers (ie, abnormal values). Navigation: clicking the warning should lead users to a section containing similar information to the available input explanation concept (Version C). In the absence of a warning, the section should contain the text “No Input Warning”; the section should not be empty. Presentation of the exact values of the parameter triggering the warning is not important.	Appreciated the explanation concept. Navigation: clicking on the warning should lead directly to a section where raw values are displayed. Requested a mechanism to acknowledge the warning and a clear distinction between new and acknowledged warnings. Presentation of the exact values of the parameter triggering the warning is important. Interaction: requested the ability to correct erroneous input values manually. In the absence of a warning, the section should be empty.
Available input	Structure: The explanation concept should be presented in an individual section of the interface and not at XUI level 2.	—

^a^XUI: explanation user interface.

#### Evaluation: XUI Level 2

##### Qualitative Analysis of Group Discussions

Overall, for the second level of the XUI, both the physician group and the nurse group had no problem identifying where the explanation concepts were presented and how to switch between them. However, 1 ICU nurse thought clicking on the recommendation would show an explanation text containing a detailed description of that specific recommended parameter.

The explanation concepts organize the following group discussion results.

##### Available Input

The nurses and physicians answered the questions AI-1, AI-2, and AI-3 correctly. Both groups correctly understood that the explanation concept informs which input parameters were available to the CDSS when it generated its recommendation (AI-1), identified in which sections input parameters were missing (AI-2), and could describe how to access more detailed information about the input values (AI-3). The physicians generally preferred more detail, while nurses favored a more concise presentation, which was expressed in the group discussions, the free-text answers, and the selection of variants.

For question AI-4, which addressed parameter ordering, the physician group expressed a unanimous preference for the static ordering used in the mockup. To avoid redundancy and reduce cognitive load, this question was omitted from the nurse session—while still ensuring space for user-specific feedback. Detailed findings are provided in Table 3.

**Table 3:** Summary of usability walkthrough insights for the “available input” explanation concept.

Question	Physician walkthrough	Nurse walkthrough
AI^a^-4	Preferred the static ordering of categories and parameters as presented in the mockup.	Not discussed
Selected version	Version C (most information).	Version A (most concise)
Discussed insights	Physicians should be deciding whether the values are outdated or not, and not the CDSS^b^ The value of the parameter triggering an “outlier warning” should be highlighted.	The other versions were seen as containing too much information. Preferred less information on the screens.
Free-text answers	None.	“Minimalize not important, not relevant data, or information.” [P5] “Deviations should be made visible.” [P5] “More is less.” [P5] “Too much information at once —> just the current errors —> one should be able to think that only the most recent blood gas analysis results would be considered [P6] Red for highlighting is okay.” [P6] “Too much information.” [P7] “Not too much information! Keep it simple.” [P8]

^a^AI-4: item 4 for group discussion of the available input explanation, as provided in Table 1.

^b^CDSS: clinical decision support system.

##### Feature Importance

The nurse and physician groups correctly answered questions—FI-1, FI-2, and FI-3—corresponding to the feature importance explanation concept. They correctly noted that the explanation concept presented the most influential factors for the recommendation (FI-1), identified which parameter had an importance of 80% (FI-2), and recognized where to click to display more parameters in the explanation (FI-3). Both groups selected Version A as their favorite. The nurses mentioned that the nursing staff would likely not use this explanation concept in clinical practice. Detailed insights for this explanation concept are provided in Table 4.

**Table 4:** Insights from both group usability walkthroughs for the explanation concept: feature importance.

Question	Physician walkthrough	Nurse walkthrough
Selected version	Version A.	Version A.
Discussed insights	Version C was viewed as beneficial for research, but not appropriate for daily clinical workflows. X-axis labels: What format would be most intuitive for clinicians? X-Axis labels: the group suggested empty labels; exact values were deemed less relevant than the relative differences between features. Participants questioned how the explanation would be interpreted or acted upon in clinical practice.	Version C would not be interesting for the nursing staff. Suspected feature importance would not be used in daily clinical practice.
Free-text answers	“Importance is not clear. Which consequences should be drawn?” [P3]	“Only if someone would like to see complete information, not to be shown as default information, only shown on demand.” [P5] “Not relevant for the daily clinical working process.” [P6]

##### Rule-Based

The physicians unanimously voiced strong concerns about this explanation concept. They feared the concept would lead to an oversimplified perception of the AI model and might mislead nonexperts. They recommended excluding this explanation from the final XUI design. Due to the physicians’ strong objections, the explanation concept was not shown to the nurses. Notes from the discussion are reported in Table 5.

**Table 5:** Insights from both group usability walkthroughs for the explanation concept: rule-based.

Question	Physician walkthrough
R-1	Correct answer.
Selected version	Version A.
Discussed insights	Group consensus: the explanation concept should not be part of the XUI^a^ for this specific CDSS^b^ for the following reasons: (1) would suggest to the user a too simple model of the system; (2) users with a low level of expertise could learn rules from the system that are not correct or clinically validated, without knowing this; and (3) an explanation that the rules, are a approximation of the system, would occupy additional resources.
Free-text answers	“Leave [this concept] out.” [P1] “False security.” [P2] “Relationships from the menu cannot be established in this way. Possibly conveys false security, false or unproven connections.” [P3] “Creates a ‘false’ sense of security or being able to understand the model, but only represents it incompletely.” [P4]

^a^XUI: explanation user interface.

^b^CDSS: clinical decision support system.

##### Final Group Discussions of the XUI

In the final group discussion, both groups agreed that no explanation concepts should be added to the XUI. The nurse group reiterated their preference for using only the first level of the XUI, suggesting that users with greater interest could optionally access Level 2. The nurses emphasized that the interface should minimize screen space usage.

### Quantitative Data Analysis

**Figure 7:**
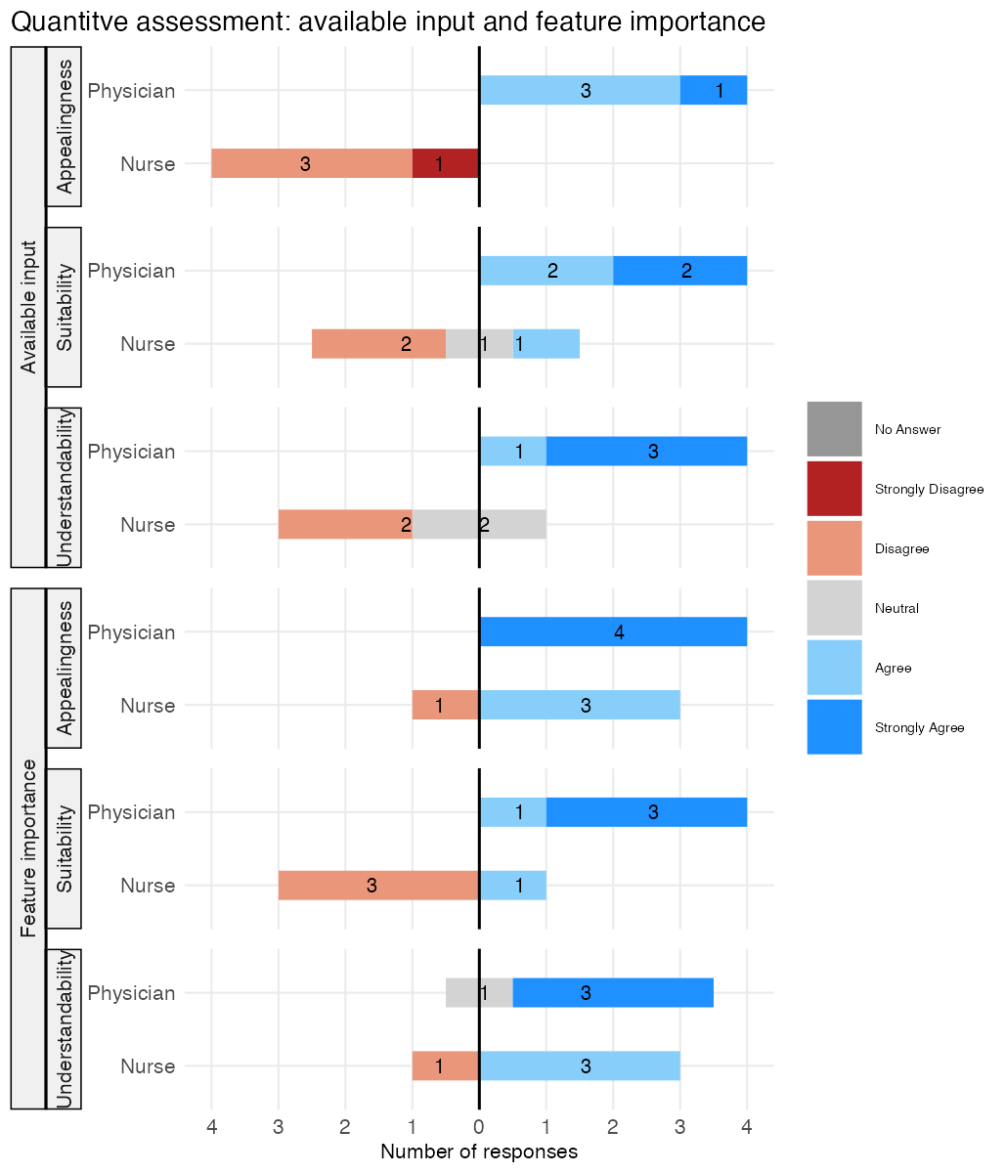
Results of quantitative assessments (QA-1 to QA-3) for the explanation concepts: available input and feature importance.

Figure 7 provides the QA results (QA-1 to QA-3) for the available input and feature importance concepts.

As shown in the top half of Figure 7, the physicians agreed or strongly agreed that the available input concept was understandable, suitable for clinical work, and visually appealing. The nurses mostly disagreed or responded neutrally regarding the concept’s suitability and understandability, and found the visualization aesthetically unappealing. The nurses’ and physicians’ ratings, therefore, differ across all assessed dimensions, with nurses being more critical overall.

The QA results for the feature importance explanation concept are shown in the bottom half of Figure 7. Most physicians strongly agreed that the feature importance concept was appealing, suitable, and easy to understand. The nurses mostly agreed with its understandability and appeal, but tended to disagree with its suitability for clinical work. Both user groups, therefore, rate the understandability and visual appeal of the explanation concepts positively. However, the user groups differ in their assessment of the suitability for their clinical work, with a positive assessment by the physicians and a negative assessment by the nurses.

**Figure 8:**
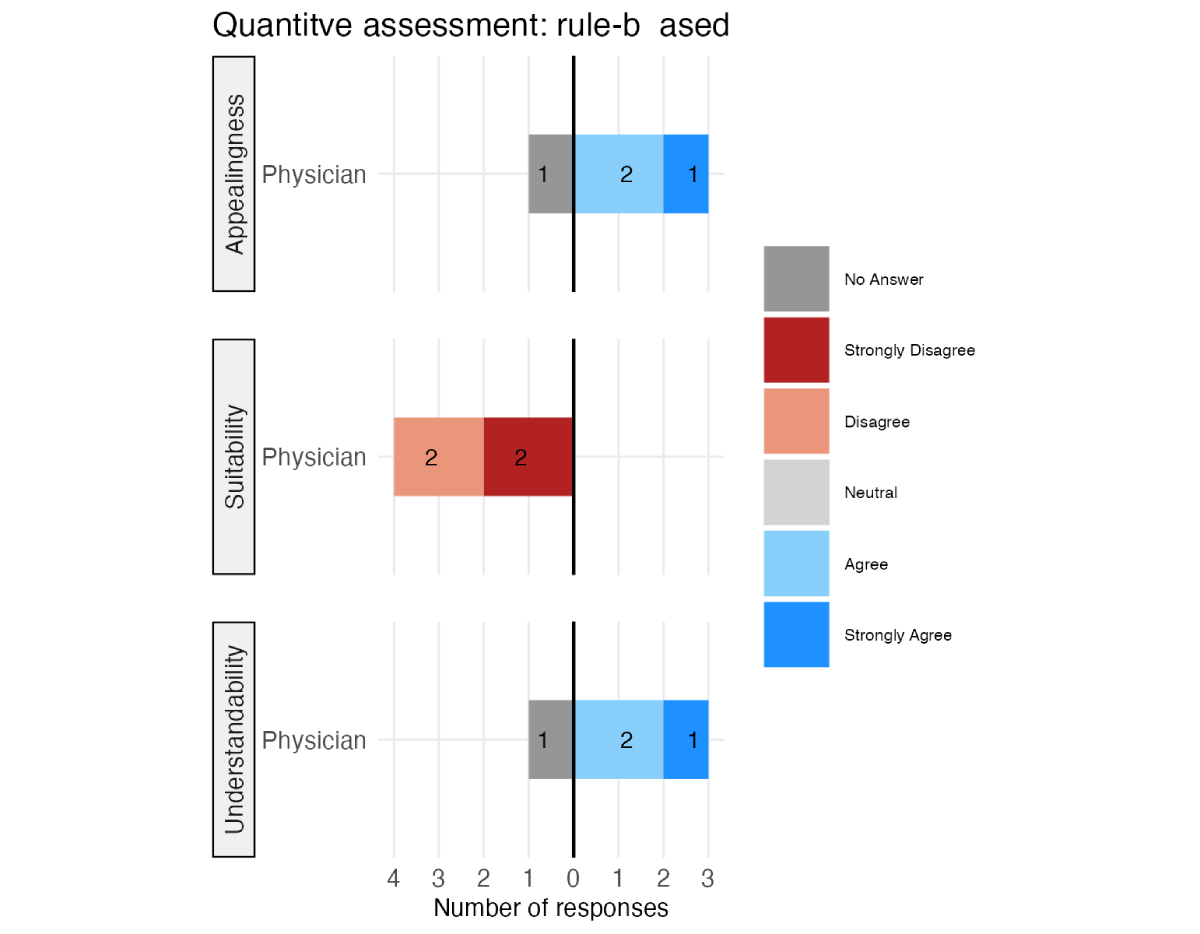
Quantitative assessment results of the rule-based explanation concept.

Figure 8 provides the QA results for the rule-based explanation concept. Three physicians strongly agreed or agreed with its appeal and understandability; one chose the “no answer” option. All 4 physicians disagreed or strongly disagreed with its suitability for clinical practice.

The calculated descriptive statistics for each QA for all 3 explanation concepts on the second level are provided in Multimedia Appendix 3. The descriptive statistics mirror the results visible in Figures 7 and 8.

## Discussion

### Principal Findings

This paper provides findings from the first design and formative evaluation phase of an XUI for an AI-based CDSS aimed at optimizing mechanical ventilation in the ICU.

The design led to an XUI with 2 levels. Level 1 of the XUI, positioned next to the CDSS recommendations on the main screen, includes the output certainty and outlier warning concepts. Level 2 of the XUI provides on-demand access to additional explanation concepts: available input, feature importance, and rule-based explanation. Multiple versions of the explanation concepts on the second level have been designed and evaluated.

The initial evaluation consisted of 2 group usability walkthroughs with ICU physicians and nurses. Both user groups liked the first level of the XUI. The physicians were satisfied with XUI level 2 but provided valuable feedback for improving it. The nurses were skeptical about whether they would use level 2 of the XUI in clinical practice.

### XUI Design Phase

For the design phase, the following research question was investigated: RQ1: “How should the XUI of an AI-based CDSS for mechanical ventilation optimization in the ICU be designed?”

To address this research question, the first focus was on the XUI structure. The 2-level structure of the XUI allowed the provision of easy-to-grasp explanation concepts next to the CDSS recommendation and more complex and more detailed explanation concepts on the second level, on demand. This structure was based on the input of the previous UCD design phases and current design recommendations [35]. This design choice aligns with new recommendations published as a preprint after the work reported in this paper was concluded. The preprint recommends multiple levels with increasing detail for XUIs for the ICU [29].

After the structure of the XUI was created, the investigation addressed the presentation of the explanation concepts themselves. The individual visualizations for the explanation concepts are comparable to typical visualizations found for XUIs in the medical domain [43,44]. The explanation concepts were kept in line with the design of XUIs to provide clinicians with an impression of the possibility of explanations at the current state of the art of research in the field.

The different versions of the explanation concepts in level 2 of the XUI varied in the level of detail presented to determine users’ preferred level of detail for this CDSS. Based on the literature, this is considered an important design decision [29,35]. Minor adaptations were made to incorporate design recommendations for the UCD of XUIs; for example, the explanation concept of feature importance was presented to the user under the term “parameter importance,” as the term feature importance was assumed not to be self-explanatory for non-AI experts. In addition, to add some variety to the different presented explanation concepts, the typical bar plot visualization for the feature importance explanation concept was extended by a version inspired by the works of Bienefeld et al [45], which created a heat map–style feature importance history in an XUI.

### XUI Evaluation Phase

During the evaluation phase, the following research question, RQ2, was investigated: “What are clinicians’ perceptions of the proposed XUI of the AI-based CDSS for mechanical ventilation optimization?”

The users’ first impression of the 2-level structure of the XUI suggests that this structure is the right approach for this CDSS. Both groups were content with level 1 of the XUI, and the physician group was satisfied with level 2 of the XUI, while the nurses were more skeptical of their intentions to use level 2. No concerns were raised by either group about the general structure. The structure allows both user groups to access the information they prefer while preventing information clutter on the main screen of the CDSS.

The combined results from group discussions, concept selection, QAs, and free-text responses indicate that ICU nurses and physicians had noticeably different preferences regarding the level of detail and complexity in the XUI. The nurses preferred less complex explanation concepts, such as output certainty and outlier warning, explicitly requested a lower level of detail for the explained concepts, and rated the suitability of the explanation concepts on level 2 lower than the physicians. The literature supports this, as it has been reported that nurses wished for clear and concise explanations, outputs, and actionable information when dealing with AI [31,46]. In contrast, the physicians were satisfied with both XUI levels, which allowed them to consult low-detail and less complex information on level 1 and more detailed and complex explanation concepts on level 2 of the XUI. This implies that the explanation needs and preferences between the nurses and physicians differ, with physicians having a higher need for more complex and detailed explanations than the ICU nurses. The work from Barda et al [31] found a similar insight. Current research supports this by showing that different health care provider groups can have different explanation preferences [15] or that different stakeholders have different explanation needs [47]. The observed differences may reflect role-specific workflows in ventilator care. ICU nurses typically operate in fast-paced bedside routines with frequent monitoring and rapid adjustments, which may favor concise, immediately actionable explanations. Physicians, in contrast, often integrate XAI outputs into broader diagnostic reasoning and decision validation, which may increase the perceived value of more detailed, mechanism-oriented explanations provided on level 2.

Notably, divergence between groups was most consistent for clinical suitability rather than for understandability or visual appeal (Figure 7). This suggests that acceptance may not only be a matter of whether clinicians can comprehend an explanation and perceive it as visually appealing, but also whether it fits their real-world task context and decision-making responsibilities. Therefore, suitability should be treated as a primary design target in XUI development, alongside clarity and aesthetics.

These results emphasize the need for a rigorous application of the UCD process when an XUI is developed for different health care providers. In the XUI developed and evaluated in this study, the 2-level solution appears to meet the differing explanation needs and preferences of the user groups. Future iterations should evaluate whether this approach remains sufficient or if separate XUIs are needed. It should be kept in mind that ICU nurses will probably not consult level 2 of the XUI in future iterations. Therefore, presenting potentially necessary and valuable information only on level 2 should be avoided. Clinical role alone may not fully explain the differences; AI literacy and ventilator-care experience likely act as moderators that shape how much explanation depth is perceived as useful versus burdensome. Future work should disentangle professional role effects from training- and experience-related factors, for example, by stratifying participants accordingly.

Interestingly, both groups were skeptical about their peers’ adaptations of the explanation concepts in the clinical daily routine, which was voiced during the group discussions and in the free-text answers. The nurse group voiced more skepticism than the physician group, and their skepticism increased with the level of detail and complexity of the explanation concepts. The physicians were mainly satisfied with the proposed explanation concepts, apart from the rule-based explanation concept. However, they stressed that the complexity of the explanation concepts should not exceed that used in the XUI. These results explain why both groups had no interest in more complex explanation concepts, such as a counterfactual explanation. This result contrasts with the results of a study by Jung et al [48], in which clinicians had a positive perception of counterfactual explanations.

The physician group positively assessed the explanation concepts on level 2 of the XUI, apart from the suitability of the rule-based explanations. The understandability ratings for the explanation concepts are similar to previous research, showing that physicians can understand the selected concepts [32]. The positive assessment of the feature importance explanation concept for the physician group is in line with other research efforts [14,15]. Due to the overall positive assessment by the physicians of both levels of the XUI and nurses’ positive perception of level 1 of the XUI, it seems that the results of the first design phase were a step in the right direction. However, the evaluation also revealed some improvement opportunities.

Second, the evaluation phase investigated research question RQ-3: “What XUI design improvements should be addressed in subsequent design iterations?”

Based on the results of the evaluation, all the explanation concepts should be revised, keeping the nurses’ preferences for a low level of detail and complexity for the explanation concept, and their perception of the explanation concept as having too much detail in mind. In the following design iteration, all the explanation concept versions should be provided with a lower level of detail or complexity as a default version. From these default versions, the more interested user should be able to access a more detailed version on demand. This approach has to be balanced so that the more interested users are not burdened while accessing the explanation concepts with the current level of detail and complexity for which they provided positive feedback in the evaluation.

For the feature importance explanation concept, the evaluation revealed that further research is needed for the design of the x-axis. The x-axis should be redesigned to be understandable for physicians and nurses and to transport the appropriate amount of information without overwhelming them. As raised in the physician group walkthrough, one option could be an empty x-axis; the suitability of such a version should be tested in comparison to other typical variants of the x-axis for this explanation concept. As the question about the actionability of the information provided in the feature importance explanation concept arose in the physician group, it should be investigated whether incorporating information about how to act on the explanation concept would be valuable. For the outlier warning explanation concept, the evaluation revealed different preferences for the case in which no outlier would be detected. It should be investigated how the user groups would react to the version they did not prefer when interacting with a version of the XUI.

An increased effort should be put into the design of level 1 of the XUI. No alternative versions had been designed for the explanation concepts on level 1 for this initial prototype to reduce complexity. These should be a primary focus for the next design iteration, as both user groups saw them as valuable. While refining these explanation concepts, an emphasis should be placed on concise information visualizations and effortless information retention from both user groups.

### Limitations

This first iteration of the design and evaluation phase of the UCD process for the XUI of a CDSS has some limitations that influence the generalizability of the results and should be carefully considered when interpreting the results of this study. A pragmatic group usability walkthrough was selected for the evaluation phase due to the limited number of available and suitable participants per user group. Nonetheless, this pragmatic approach proved valuable as it allowed for the receipt of early feedback from subject matter experts on mechanical ventilation with real-world and research experience. Their experience might have biased their assessment of the XUI, as they might not have had to rely on the CDSS in the first place due to their extensive experience. However, their knowledge allowed them to focus on the XUI design and incorporate extensive practical knowledge and expertise in their feedback and assessment of the XUI. The small group size of only 4 participants per group usability walkthrough might be seen as a limitation, but this group size allowed for a lively discussion between participants, and smaller sample sizes are in line with early evaluation iterations [40,41]. In addition, the group walkthrough allowed for the collection of feedback in a condensed amount of time, which was necessary due to resource restrictions. Nonetheless, these points limit the generalizability to the clinical population that should be addressed in the following design iterations.

Furthermore, the changes for the nurse walkthrough limit the comparability between the 2 walkthrough groups. The walkthrough had to be adjusted to avoid overwhelming the participants. Despite the minor adjustments, the collected insights allow for a preliminary assessment of the difference between the user groups. No actual data or patient vignettes were used to promote deeper engagement or trigger demand for an explanation from the CDSS, which limits the comparability of the assessment of XUI in real-world situations. The current state of the XUI mockup was sufficient to spark extensive discussions while preventing the participants from focusing on the medical validity of the situation or mockup content. In this initial iteration of the UCD, only subjective assessments of the XUIs were elicited. This may not provide a complete picture, as it has been shown for XUIs in the ICU that subjective assessments might not transfer to the use or influence of the XUI during the medical decision-making process [30,49]. Nevertheless, the amount of valuable insights generated through subjective assessments of the XUI in this study shows that this type of feedback is helpful during such an early design phase. These limitations should be addressed in future evaluation iterations.

### Future Research

Based on this research, the XUI mockup should be improved. The improvements should address the identified opportunities for enhancement in the XUI, making it more suitable for both user groups without drastically altering it, as physicians were already satisfied with the current design. A higher focus should be placed on level 1 of the XUI, as this is likely the most used part. In addition, feedback should be sought from the AI and XAI algorithm developer and incorporated into the mockup to enhance the realism of the XUI for the next iteration. An improved and extended XUI mockup would be a suitable basis for a more elaborate evaluation. The enhanced mockup could be accompanied by different vignettes of patients and situations that might trigger the need for an explanation from the user. Future evaluation rounds should have a higher experimental character to avoid the potential shortcomings of subjective assessments of an XUI in the ICU and allow the users to interact with the XUI directly. The proposed evaluation iterations should aim for a higher sample size and a diversified participant pool concerning their ventilation experience, AI literacy, and clinical center to foster generalizability and reflectiveness of the clinician population. To this end, a remote experimental study could be conducted in which the user directly interacts with the CDSS based on presented patient vignettes. During such an evaluation, participants reflecting a broad base of clinical users should be recruited from multiple institutions.

### Conclusion

To the best of our knowledge, this paper is the first to report on the UCD and formative evaluation of an XUI for an AI-based CDSS aimed at optimizing continuous mechanical ventilation in the ICU. The findings indicate that a 2-level XUI structure can effectively address the distinct explanation needs of ICU clinicians. Nurses’ information needs were largely met through concise, high-level explanations such as outlier warning and output certainty, presented alongside CDSS recommendations, whereas physicians preferred access to more detailed explanations—such as available input and feature importance—on a secondary level. These results provide empirical evidence that explanation needs differ by professional role and underscore the necessity of user-centered, iterative design processes in the development of XAI systems for critical care. Future work should refine these explanation concepts and evaluate their impact on clinical decision-making, user trust, and system adoption.

## Appendix

Multimedia Appendix 1 STARE-HI checklist.

Multimedia Appendix 2 Quantitative assessment sheet.

Multimedia Appendix 3 Descriptive statistics for quantitative assessments of each explanation per user group.

## Abbreviations

AI: artificial intelligence
CDSS: clinical decision support system
DIN: Deutsches Institut für Normung
EN: Europäische Norm
EU: European Union
FI: feature importance
ICU: intensive care unit
ISO: International Organization for Standardization
QA: quantitative assessment
STARE-HI: Statement on the Reporting of Evaluation studies in Health Informatics
UCD: user-centered design
XAI: explainable artificial intelligence
XUI: explanation user interface
