# Supplementary material for: An Explanation User Interface for Artificial Intelligence–Supported Mechanical Ventilation Optimization for Clinicians: User-Centered Design and Formative Usability Study
Source: JMIR Form Res. 2026 Feb 3;10:e77481. doi: 10.2196/77481 (PMC12914239; doi:10.2196/77481)
Supplement: Multimedia Appendix 2 [file formative_v10i1e77481_app2.pdf]

(Translated from German to English for publication.)

Evaluation of the IntelliLung-DSS Explanation User Interface

Explanation Concept 1: **Available Input**

**I find this explanation concept …**	Strongly Disagree	Disagree	Neutral	Agree	Strongly Agree	No Answer
… easy to understand.						
… suitable for everyday clinical work.						
… appealingly visualized.						

Free text comment:

Explanation Concept 2: **Parameter Importance**

**I find this explanation concept …**	Strongly Disagree	Disagree	Neutral	Agree	Strongly Agree	No Answer
… easy to understand.						
… suitable for everyday clinical work.						
… appealingly visualized.						

Free text comment:

Explanation Concept 3: **Rules**

**I find this explanation concept …**	Strongly Disagree	Disagree	Neutral	Agree	Strongly Agree	No Answer
… easy to understand.						
… suitable for everyday clinical work.						
… appealingly visualized.						

Free text comment:
