# Supplementary material for: An Explanation User Interface for Artificial Intelligence–Supported Mechanical Ventilation Optimization for Clinicians: User-Centered Design and Formative Usability Study
Source: JMIR Form Res. 2026 Feb 3;10:e77481. doi: 10.2196/77481 (PMC12914239; doi:10.2196/77481)
Supplement: Multimedia Appendix 3 [file formative_v10i1e77481_app3.docx]

**Table 6**. Descriptive statistics for quantitative assessments of each explanation per user group.

|  | Understandability | | Suitability | | Appealingness | |
| --- | --- | --- | --- | --- | --- | --- |
|  | Physician Group | Nurse Group | Physician Group | Nurse Group | Physician Group | Nurse Group |
| **Available input** | | | | | | |
| No of responses | 4 | 4 | 4 | 4 | 4 | 4 |
| No of “No Answer” | 0 | 0 | 0 | 0 | 0 | 0 |
| Median | 5 | 2.5 | 4.5 | 2.5 | 4 | 2 |
| IQR | 0.5 | 1 | 1 | 1.5 | 0.5 | 0.5 |
| **Feature importance** | | | | | | |
| No of responses | 4 | 4 | 4 | 4 | 4 | 4 |
| No of “No Answer” | 0 | 0 | 0 | 0 | 0 | 0 |
| Median | 5 | 4 | 5 | 2 | 5 | 4 |
| IQR | 1 | 1 | 0.5 | 1 | 0 | 1 |
| **Rule-based** | | | | | | |
| No of responses | 3 | Not presented | 4 | Not presented | 3 | Not presented |
| No of “No Answer” | 1 |  | 0 |  | 1 |  |
| Median | 4 |  | 1.5 |  | 4 |  |
| IQR | 1 |  | 1 |  | 1 |  |
